# Animal Production With Restrictive Use of Antibiotics to Contain Antimicrobial Resistance in Sweden—A Qualitative Study

**DOI:** 10.3389/fvets.2020.619030

**Published:** 2021-01-15

**Authors:** Ingeborg Björkman, Marta Röing, Susanna Sternberg Lewerin, Cecilia Stålsby Lundborg, Jaran Eriksen

**Affiliations:** ^1^Department of Public Health and Caring Sciences, Health Services Research, Uppsala University, Uppsala, Sweden; ^2^Department of Biomedical Sciences and Veterinary Public Health, Swedish University of Agricultural Sciences, Uppsala, Sweden; ^3^Department of Global Public Health – Health Systems and Policy (HSP): Improving the Use of Medicines, Karolinska Institutet, Stockholm, Sweden; ^4^Unit of Infectious Diseases, Department of Clinical Science and Education, Södersjukhuset, Karolinska Institutet, Venhälsan, Södersjukhuset, Stockholm, Sweden

**Keywords:** containing antibiotic resistance, one health, animal production, poultry, Sweden, qualitative study

## Abstract

Antibacterial resistance (ABR), is a growing global threat to human and animal health. Efforts to contain ABR are urgently needed. This qualitative interview study explored perceptions of work to contain ABR among stakeholders in food animal production in Sweden, with focus on broiler production. Semi-structured interviews were carried out with a strategic sample of 13 stakeholders in different parts of production, from professionals at policy level, veterinary authorities, to poultry farmers and poultry veterinarians. Conventional inductive content analysis was used for data analysis. A latent theme, “Working in unison,” emerged, based on the consistency expressed by the informants when they discussed ABR, use of antibiotics, and animal health management. This theme was built on four domains representing the content of the interviews: Knowledge and engagement; Cooperation; Animal health concept; and Development in balance with economic prerequisites. According to the informants, ABR has not been an isolated issue in Sweden but has been included in a tradition of animal health and welfare, and actions have been driven by the industry or by government regulations. Veterinarians described how they worked closely with farmers. Farmers felt involved in the development of animal health management methods. The One Health concept was well-known among stakeholders at national level but not at farm level. Close cooperation between stakeholders seems to facilitate development of animal production with low use of antibiotics.

## Introduction

Antibacterial resistance (ABR), is a growing global threat to human and animal health (1, 2). In 2013 ABR was highlighted as one of three global risk cases (3). ABR is not slowing down (4), and efforts to contain ABR are still urgently needed. In 2015 the World Health Organization (WHO) announced a Global Action Plan based on a “One Health” approach (5). One Health implies collaborative efforts between stakeholders at different levels in sectors working with animals, humans, and environment. The global action plan emphasizes a need for coordination between international sectors and actors including human and veterinary medicine, agriculture, environment, finance, and consumers (5). This approach is important since resistant bacteria can be transmitted between humans, animals, and the environment, and across international borders.

Efforts to contain ABR began early in Sweden. In the human health sector, the Swedish strategic programme against ABR, “Strama,” was formed in 1995 (6). Even earlier, in 1986, the use of antibiotics for growth promotion in animals was banned (7). When Sweden joined the European Union in 1995, extensive lobbying contributed to the current EU-wide ban on antimicrobial growth promoters (8, 9). From an international perspective, current levels of antibiotic use in animals (10) and the prevalence of ABR in bacteria from animals (11) in Sweden are very low. Veterinarians are not allowed to make a profit from selling drugs, and antibiotics for animals can only be obtained in registered pharmacies, based on veterinary prescription. Regulations on veterinary medicines also restrict which antibiotics may be prescribed. The European Medicines Agency category A substances (12) are not allowed while category B substances may only be prescribed when absolutely necessary, i.e., when demonstrated by bacterial culture and susceptibility testing.

### Swedish Broiler Industry

Antibiotics are rarely used in Swedish broiler production. The Swedish broiler industry is quite homogenous. Some large production companies own or have franchising contracts with most farmers and slaughterhouses while a few actors have independent contracts along the production chain, from breeding to slaughter. Genetic material is imported in the form of day-old chicks from one large international company based in the United Kingdom. The Swedish Poultry Meat Association (“Svensk Fågel”) organizes the majority of all production companies, slaughterhouses and individual farmers. While the Swedish Poultry Meat Association is a lobby organization for Swedish broiler production, it also conducts monitoring and control programmes for animal welfare and productivity, cocciodiosis and clostridiosis, *Salmonella* spp. and *Campylobacter* spp., as well as for antimicrobial use. One veterinarian employed by the association is responsible for strategic work with programmes that cover >98% of all broilers in commercial production. Coccidiostats are prescribed when needed, as part of the cocciodiosis control programme, with narasin being the most common substance (11). There is only a small number of organic broiler farms. The production of broilers has increased from 78 million in 2010 to 100 million in 2018 (13). Only four out of 3223 flocks (0.12%) were treated with antibiotics in 2018, a level that has been similar for several years. However, an increase was noted in 2019, mainly due to necrotic enteritis (a clostridial infection that can be controlled by coccidiostats) (11).

### From Strategies Into Action

The Swedish government strategy for containing ABR from 2016 takes a One Health approach with the overall goal to preserve the possibility of effective treatment of bacterial infections in both humans and animals (14). The strategy was updated in 2017 and again in 2020 (15). The Public Health Agency of Sweden and the Swedish Board of Agriculture jointly coordinate the work against ABR and healthcare-associated infections.

In order to contain ABR, knowledge and social engagement, as well as action from different levels of society are needed. Although knowledge is available, actions are still insufficient, and the ABR problem is growing at global level (2). It is therefore important for countries to study the process of how knowledge and action plans are transformed to practice, so they can learn from each other and speed up this process.

This study is part of the ABRCARRO (A One Health Systems and Policy Approach to Antibiotic Resistance Containment: Coordination, Accountability, Resourcing, Regulation and Ownership)—an international project which aims to explore and describe how national action plans against ABR were developed, implemented, monitored and evaluated in Sweden, South Africa and Swaziland. The project includes interviews with different categories of stakeholders, at government level, for example policymakers, and professionals in human, animal, and environment/agriculture sectors, as well as policy document analyses. The aim of the present study was to describe how Swedish stakeholders in animal production, with a specific focus on poultry, perceived efforts to contain ABR.

## Methods

### A Qualitative Design

To explore the views of stakeholders in animal production a qualitative design was chosen. A qualitative design can give new understanding about social events in areas where knowledge is limited (16, 17). Data is often collected in interviews with persons who have experience of the topic in question (18). A focus on broiler production was chosen, as the homogeneous structure of this industry made stakeholders easy to identify. Furthermore, it was expected that sufficient data presenting the reality of this industry could be gained with a limited number of participants. In addition, intensive poultry production has more similarities worldwide than most other livestock sectors (19). A strategic sample of informants was recruited with the purpose of capturing perceptions of stakeholders from different parts of the production, with their unique perspectives. Informants were professionals at policy level, from veterinary authorities or industry organizations, and field actors such as poultry farmers and poultry veterinarians.

A total of 13 stakeholders were interviewed, see Table 1. All informants at policy level and from the animal industry organizations were veterinarians. Sweden is a small country, and the nine veterinarians represented approximately one third of the professionals responsible for ABR issues in the livestock sector at national level. The farmers constituted only a small number of all broiler producers in Sweden. One layer producer was added to widen the farmer perspective. All interviews were carried out by one of the authors (IB) between January and June 2018. The interviews lasted between 40 and 96 min, on average 62 min. Policy informants and informants from industry organizations were contacted via email, informed of the purpose of the study, and asked to participate. Snowballing was used to find farm-level actors, veterinarians as well as farmers. The layer farmer was recruited via telephone contact. Informed consent was obtained in writing from all informants.

**Table 1:** Description of informants.

**Level**	**Informants**
Policy level (3 informants)	Two veterinarians working at the National veterinary institute; one expert on antibiotic resistance and one expert on ruminant diseases. One veterinarian responsible for antibiotic resistance issues in the Swedish Board of Agriculture
Industry organizations (3 informants)	Head veterinarian in the Swedish Poultry Meat Association. Head veterinarian in the Swedish Egg Producers' Association. Head veterinarian in The Federation of Swedish Farmers
Farm-level actors (3 + 4 informants)	Three poultry veterinarians employed by broiler companies. Three broiler farmers and one layer farmer

### Interview Guide

A semi-structured interview guide was developed, the main questions are listed in Table 2. The questions were based on an interview guide previously used by the research group. The interview guide was piloted on two informants, one from the animal sector and one from the human sector [the human sector study is presented elsewhere (20)]. The pilot test did not indicate any need for changing the interview guide and therefore the pilot informants were included as participants in the respective studies. The interviews were performed at a place convenient for the informants, often their workplace. The informants could associate and speak freely from the main questions, and the interviewer followed the conversation and asked probing questions. All interviews were audio-recorded and transcribed *ad verbatim* by an external transcriber. Before further analysis, the first author listened to all recordings and checked all transcripts.

**Table 2:** Interview guide used for interviews, main questions.

1. What does antibiotic resistance mean to you?
2. How do you see your role in working to contain antibiotic resistance?
3. How do you see possibilities of limiting/preventing emergence and spread of antibiotic resistance?
4. What do you think are the main causes of antibiotic resistance?
5. How do you think antibiotic resistance is spread?
6. How do you see the use of antibiotics in humans, animals, or any other areas?
7. Have you heard of the concept of “One Health”?
8. Do you have any comments to add?

### Analysis

The first author (IB) analyzed all interviews. No theories or predefinitions were used, and conventional inductive content analysis was chosen (21). Initially, two of the authors (IB and MR) read the same transcript and marked meaning units and wrote preliminary codes. Then the researchers met, discussed, and agreed on how to proceed with the analyses. A first scheme of codes was constructed. Then the texts were processed line by line and meaning units were picked using the scheme of codes, which were used to sort the content from all interviews. In a next step the content of the codes was condensed, and codes were grouped in comprehensive categories. Next the codes were condensed again, rearranged, and merged. During this process, a latent theme built on four domains emerged. During the analysis IB and MR met several times and discussed the process and findings. In a final step all researchers discussed and agreed on the findings.

## Findings

The informants in this qualitative interview study (policymakers, poultry farmers, and poultry veterinarians), despite expressing their thoughts in different ways, were very much in agreement and shared a similar picture of work to contain ABR. The latent theme emerging through the analysis, and labeled “Working in unison,” reflects this agreement. This latent theme was found in four domains and categories, which represent the manifest content of the interviews. The relation between theme, domains and categories is shown in Table 3, and each of these categories is described in the text below. In Table 4, quotes from the informants are sorted in the different domains. The interviewed veterinarians often had similar opinions, irrespective of professional position. These similar thoughts are summarized and presented collectively under “the veterinarians.” If and when veterinarians differed in their opinions, their respective positions were described. In general, the veterinarians provided more comprehensive statements and more details than the farmers, especially in theoretical issues, but the farmers' opinions and knowledge were in line with those of the veterinarians. The farmers gave detailed information on how they worked and why.

**Table 3:** Domains and categories under the latent theme identified in the interviews.

**Theme**	**Working in unison**			
Domain	Knowledge and engagement	Cooperation	Animal health concept	Development in balance with economic prerequisites
Categories	- Perceptions of antibiotics and antibiotic resistance - Necessary to contain antibiotic resistance - Reduce the use of antibiotics - Awareness needed	- Cooperation a key factor - One Health	- Healthy animals do not need antibiotics - Low use of antibiotics for animals - Infection control to promote animal health - Long tradition of national efforts	- Broiler production in Sweden is large scale and controlled at central level - Conditions and management in Sweden differ from many other countries - Economy rules food production

**Table 4:** Quotes from all of the informants sorted in domains.

**Domain**	**Quotes**
Knowledge and engagement	We have two possible tools we can work with - wise antibiotic use, and we can work with preventing infections. And then it is not only the spread of resistant bacteria, but all kinds of infections. […] As it looks today, we can't **afford** not to work with both tools, and I don't believe, I don't believe it will be as effective if we don't work with both. *Policymaker informant 1*
	So we try, oh, oh…yes…to keep discussions alive during the whole year, both about disease control but above all the use of antibiotics in this area. *Industry informant 1*
	When I write texts about this, I rarely need to change much if I publish it in the agricultural press or veterinary press. Because we both have great knowledge, and we are well aware of the issue. *Policymaker informant 2*
	But we try to work in such a way so that we don't use it [antibiotics], because - actually we think somehow that…it is not necessary [in chicken production] - it is instead very much about management factors. *Poultry veterinarian 3*
	So all the breeders really work to minimize the risk of contamination in the stable. So we change clothes completely and yes, or… you have done something so you wash your hands once again if you want, but now it is a fairly clean environment here so to speak, and then shoes are changed once more as well. *Farmer 2*
Cooperation	Everyone, everyone owns it [the antibiotic resistance issue]. And that's what I think we are so successful with in Sweden, eh, that we. If I look at the animal sector, then it is really that we veterinarians work **together** with the farmers a lot in this matter. *Policymaker informant 2*
	But I can never communicate, succeed in communicating with all Swedish veterinarians and farmers. Possibly with veterinarians, but not farmers. And they are the ones we need to reach in the end. Eh…they also need knowledge. And then one must work, must and must, but then my idea is to work via contacts, which is most effective, and to do so in close agreement with them. *Policymaker informant 1*
	In our field we have been quite skilled at cooperating with authorities, I think, and have developed a lot of these different programs to ensure the quality we have. *Farmer 1*
	Facilitating, it's, that we are so. have so much in common and cooperate, so that everyone doesn't need to do it at home in their house, but that we can actually share, so if the other company does tests to see if you can hatch chicken without this bacterium, for example, just by a very fine egg quality, they share the result so that we others can see it. *Poultry veterinarian 1*
	You have to work together, eh, so that you, as a rich western country, do not just sit on your high horse and eh, judge and point with your whole hand and say that now you should do this. On the contrary, you have to actually help. *Industry informant 2*
Animal health concept	It is very rare that I have used antibiotics during the time I have done this [chicken-meat production]. Interviewer: Mm, how long is that? Yes, it is almost 20 years, I think I can count on one hand what I have been prescribed [to the animals]. *Farmer 3*
	There is a constant struggle with maintaining biosecurity, mentality, education, new people keep coming, experienced people quit, and you have to keep the flow moving. *Poultry veterinarian 2*
	Sweden as the first country banned antibiotics as growth promoters in 1986. And then you **saw**, it wasn't just. eh, the effects were quite big, because it was not just growth promoting, that antibiotics smoothed **over** management flaws. *Industry informant 3*
	There, like, the state did not go in, but the industry decided, it was an agreement then, that farmers, veterinarians, veterinary organizations decided that we should, we should eradicate it [Bovine virus] from the country. And then it was a voluntary control program. *Policymaker informant 2*
Development in balance with economic prerequisites	We have a *Salmonella spp* control program for example, you can eat your eggs raw in Sweden, you can do quite a lot that you cannot do in other countries. And it is something that has cost money, so this has been yes, it has been done with government grants. *Policymaker informant 3*
	The risk is, if you go too fast [in changing production methods], you get setbacks and then the producer says that this is not possible, it's not possible - and then you are back where you started. *Industry informant 1*
	Yes, I think we have really good breeders, most of them. And they, they want, for their own sake, it is very much about avoiding *Salmonella*, after all, and it goes hand in hand as well. *Salmonella* and *Campylobacter* for them are the ones they work, they get deductions if they have *Campylobacter*, and [if they have] *Salmonella* so the whole flock is slaughtered. *Poultry veterinarian 3*
	Of course it is important that we are compensated for the extra cost eh, that this system in this case has cost us, and partly it is about communication with the consumer and explaining why this is a little bit more expensive yet has these advantages, and one may well need help from authorities and politicians as well as to explain and describe. *Farmer 1*

### Knowledge and Engagement

#### Perceptions of Antibiotics and ABR

All informants, except one of the farmers, were engaged in the issue of antibiotics and ABR. They shared the perception that antibiotics are needed but must be used restrictively. ABR was described as a very serious threat, leading to inability to treat bacterial diseases or to perform surgery safely, and to increased mortality. A common perception was that ABR already exists, but that the real threat is a future problem. Some of the veterinarians compared the ABR issue with the issues of environment and climate—slowly emerging threats which require behavioral change. The informants perceived ABR as caused by excessive consumption of antibiotics in the public health sector, and that the animal sector also contributes to ABR development. They felt that ABR mainly emerged abroad and was imported to Sweden.

#### Necessary to Contain Antibiotic Resistance

All veterinarians emphasized that containment of ABR was necessary, and that measures must be taken in both the animal and human sectors. Both veterinarians and farmers perceived ABR as an issue for everyone—everyone should take an interest, and authorities, as well as the entire animal production sector, should be involved. Physicians and veterinarians also need to take responsibility for not prescribing antibiotics unnecessarily, one industry veterinarian pointed out. Treatment of pets was a special issue according to some veterinarians, as animal owners may demand antibiotics for their pets, against the recommendations of the veterinarian. One policy veterinarian explained that pets nowadays are perceived as family members.

#### Reduce Use of Antibiotics

The veterinarians stated that eradicating ABR is difficult, but reducing antibiotic use is possible, and the purpose of this is to reduce selection pressure. The need for antibiotics in food-producing animals is a matter of production and management methods, veterinarians said. One poultry veterinarian used a “wait-and-see” approach instead of prescribing antibiotics and offered a second visit to the farm some days later to check up on the animals. Before choosing antibiotic treatment, the poultry veterinarians said they always took samples for bacterial culture and susceptibility testing and used narrow-spectrum antibiotics if treatment was deemed necessary. It was obvious to the farmers not to use, or rarely use, antibiotics for animals and instead practice good hygiene, disease prevention and infection control. One farmer explained that this was a daily never-ending process.

#### Awareness Is Needed

The informants expressed that to make people follow available recommendations, awareness, knowledge, and understanding were necessary. The perception was that Swedish stakeholders and the public in general were aware of ABR and that this facilitates the reduction of antibiotic use. Both veterinarians and farmers gave examples of outbreaks of infections that had increased awareness, both in Sweden and internationally. Veterinarians suggested that media can contribute to ABR awareness among the public. The farmers referred to general media and industry specific publications when they described what they knew about ABR. The informants believed that the general awareness was lower internationally than in Sweden.

### Cooperation

#### Cooperation Is a Key Factor

The informants described the close cooperation between authorities, academia, industry organizations and farmers in the animal sector in Sweden and this was considered to be a facilitating factor. Broiler farmers pointed out that they had been involved in the development of improved management methods. As one farmer explained, regulations set up by authorities without consulting poultry farmers would not work, since farmers need to understand the whole picture. Veterinarians said that knowledge on good animal health management methods was easy to spread. All actors in the poultry production chain were members of the industry organizations (Swedish Poultry Meat Association or Swedish Eggs). Poultry veterinarians said they were few in number and that they meet at the Swedish Poultry Meat Association. The broiler farmers also said they meet regularly at the Swedish Poultry Meat Association's gatherings.

One obstacle described by some veterinarians was the “blame game”. This meant blaming others for insufficient actions. This could occur between the animal and the human sector, both locally and internationally, or between countries. Such attitudes could hamper the will to collaborate and obstruct efforts to reduce antibiotic use, said the veterinarians.

#### One Health

Two policymaker informants described their engagement in the coordinating platform of the Swedish Public Health Agency and the Board of Agriculture. One of them explained that collaboration in Sweden between the animal and human sectors at policy level had been started by Strama in the 90s. The other informant thought the platform had reduced the blame game between the animal and human sectors. The informant described a new discussion on cost-sharing, i.e., that costs could be shared between the two sectors when actions were taken in the animal sector for the sake of public health.

Policymaker informants spontaneously brought up their role in One Health and industry informants said they knew the concept well. Two of the poultry veterinarians had heard about One Health, whereas the concept was unknown for the farmers. Two of the policymaker informants had insights in the issue of ABR in the environment and believed that more knowledge was necessary in order to understand the impact of this.

### Long Tradition of Animal Health Concepts in Sweden

#### Healthy Animals Do Not Need Antibiotics

A facilitating factor in efforts to contain ABR, according to one policymaker informant, was that ABR has never been looked upon as an isolated issue but as part of a whole, bigger picture. All informants highlighted that animals who are well-cared for feel better and stay healthy. “Healthy animals do not need antibiotics” was repeated by many informants as a motto. Veterinarians claimed that healthy animals have better immunological responses and are more resistant to disease.

#### Low Use of Antibiotics in Animals

All informants talked about how rarely antibiotics are used in animals in Sweden and how they did not see ABR as a problem in animal production in Sweden. Two of the farmers said they had been in the poultry industry for 20 years and had used antibiotics in total five times each during these years. One industry informant explained that laying hens are never given antibiotics. However, all informants brought up how Swedish broilers had been infected by ESBL (Extended Spectrum Betalactamase producing *Enterobacteriaceae*) from imported breeding animals in 2010. Veterinarians said ESBL was still present but had decreased among the animals since then. As one poultry veterinarian explained; *E coli* infections in chickens are not treated with antibiotics so as not to promote the spread of ESBL, instead the focus is on infection prevention. Both veterinarians and farmers reported that sick animals are culled.

Areas for improvement in the Swedish animal sector were mentioned by the veterinarians. This included reducing antibiotic treatment of pets and the use of coccidiostats in broilers. There was also a perception that veterinarians trained outside Sweden often had other views on antibiotic treatment and prescribed antibiotics more often than those who received their veterinary education in Sweden. All informants thought that antibiotics were used extensively in animal production outside Sweden. However, it was also pointed out by some veterinarians that many countries are currently working hard to change their animal production and reduce the use of antibiotics.

#### Infection Control to Promote Animal Health

All informants mentioned the importance of infection prevention. Veterinarians emphasized that this was a way to reduce the need for antibiotics and that it was of economic benefit to prevent infections. Policymaker informants and informants from the industry organizations described measures to prevent spread of infections, e.g., contact isolation, culling animals, trading only with non-infected regions, and practicing biosecurity. Poultry veterinarians and farmers gave detailed descriptions of how they worked with biosecurity, e.g., strict hygiene routines with hygiene barriers and visitor restrictions, keeping records, and standards for stables. They perceived biosecurity routines as well-established and followed by all poultry farmers. As one farmer expressed “this is how we work, all farmers in the poultry sector do it.” One industry informant pointed out that, although Sweden belongs to the common EU market, so far it has been allowed to keep stricter regulations for importation of animals and breeding material, thanks to the successful eradication of many diseases. One policymaker informant mentioned that in 2013 Sweden was the first country to launch a legislation of infection control in veterinary medicine.

#### Long Tradition of National Efforts

Veterinarians noted that Sweden has eradicated several diseases in different farm animals. Some of them explained that bovine tuberculosis was eradicated already in the 1920s, as a government initiative. One policy veterinarian gave another example, bovine viral diarrhea, and described a voluntary infection control program developed by farmers, veterinarians, and livestock industry organizations. Industry veterinarians said that in situations where vaccines cannot be used, whole-herd culling is sometimes used for disease eradication and this can be extremely hard for farmers.

Some policy and industry veterinarians mentioned the Swedish so called “Alvesta epidemic” in the 1950s, a large salmonellosis outbreak that caused the death of 90 people. Their perception was that this outbreak prompted the development of *Salmonella* control and animal welfare programs. Another important step according to the veterinarians was the banning of antibiotics for growth promotion in 1986. They noted that this initiative had been taken by farmers. According to one industry informant, when antibiotics for growth promotion were not used anymore, animal production methods had to be changed. Farmers perceived that animal welfare programs had been used for decades. Many informants wished Sweden could be a role model for other countries and show that it is possible to change livestock management systems.

### Development in Balance With Economic Prerequisites

#### Broiler Production in Sweden Is Large-Scale and Tightly Controlled

Broiler production in Sweden was described by the informants as industrial, large-scale, and well-controlled. They described how the domestic broiler industry resembled a pyramid, with a few breeding companies at the top. Hatcheries on another level in turn deliver day-old chicks to broiler farms. Farmer informants explained that the slaughterhouses plan, based on expected consumer demand for poultry meat, and calculate the number of day-old chicks to be ordered from the hatchery. To enable high biosecurity, the birds live indoors until slaughter. All participating veterinarians and broiler farmers recommended locally produced food and closed stables rather than organic production, which was regarded riskier for the birds and too costly for many consumers due to higher production costs.

#### Conditions and Management in Sweden Differ From Many Other Countries

Informants noted that production methods for Swedish broilers differ from many other countries. By “other countries” they usually meant the rest of Europe except the Nordic countries, but sometimes it included the rest of the world. For example, it was stated that the maximum bird bodyweight allowed per square meter in the stable was higher in other countries compared to Sweden. Another example was that antibiotics were not used for growth promotion, as mentioned above. Veterinarians said that all countries in the EU have a common animal legislation but, despite this, production methods and level of antibiotic use vary.

Both veterinarians and farmers talked a lot about their efforts to make the animals feel comfortable and be as healthy and strong as possible by focusing on prevention, biosecurity, and animal welfare instead of using antibiotics. One industry veterinarian concluded that changing production methods had been costly but now they see the benefits. Veterinarians said Sweden benefited from having a cooler climate with seasonal variation, and that the risk of spreading disease is higher in warmer countries. Veterinarians brought up how, in some countries, veterinarians earn their salary from selling medications, in contrast to Swedish veterinarians.

#### Economy Rules Food Production

One farmer explained the economic benefit of following all control programs very carefully, how costly it would be if you have a large production and something went wrong. One poultry veterinarian reflected that it is not laws that rule production, it is profitability. One policy veterinarian believed that in Sweden, agreements and guidelines and voluntary actions had been more important than legislation in the development of animal production methods, while another policy veterinarian believed that governmental funding had prompted this development. Both veterinarians and farmers gave examples of when the government contributed financially to control certain diseases. One lesson, according to an industry informant, was that production change must be allowed to take time, otherwise there may be backlash effects, producers may stop believing that animal production without relying on antibiotics is possible and may not want to cooperate. The economic interest of the food industry was highlighted by some informants as a possible barrier to change of production methods, as people have short-term views of economic profit.

All informants stressed that farmers must be able to live on their production, and if they cannot sell their goods, production will end. They perceived that Sweden has come far in developing a production without antibiotics, but the issue now is to be able to continue selling the products. Both veterinarians and farmers argued that buying Swedish meat supports a production that uses less antibiotics. However, they said, this production is more expensive, which generates a higher price for the consumers. Informants believed that many Swedish consumers trust the Swedish production of meat and wanted to buy Swedish, but they worried that consumers would still choose food produced abroad because of lower cost. Here, informants suggested that the Swedish government could support Swedish production by explaining to consumers why Swedish meat is more expensive. A further threat to the Swedish production mentioned by one industry informant was the Swedish animal rights organizations which seemed to work hard to destroy animal production in Sweden.

Informants from all categories acknowledged the need of resources in work to contain ABR. Veterinarians pointed out that resources and conditions differ globally, and countries may have to prioritize other measures. They expressed that work to contain ABR must continue in Sweden, but in addition Sweden and EU have an international responsibility to support the containment of ABR by funding and expertise.

## Discussion

This study gives insight into how a group of stakeholders at different levels of the animal sector in Sweden describe their work in animal production without extensive use of antibiotics, and how they believe that this has been made possible. They suggested that this is built on a long-standing culture of cooperation between stakeholders and a shared view of animal health and welfare. A latent theme “Working in unison” reflects the unity expressed by the informants when discussing ABR, use of antibiotics and production methods, with particular focus on poultry. Central findings from this study were the close cooperation between policy and industry, which included farmers as active partners in the development of production methods, with a focus on preventive measures for animal health. The improvement of animal health and welfare was based on voluntary agreements and guidelines, and also on legislation and governmental funding. According to suggestions from the informants, the Swedish government could support Swedish production by explaining to consumers why Swedish meat is more expensive. In this manner, “working in unison” can even include consumers.

Policies and action plans at global and national levels recommend restrictive antibiotic use in order to contain ABR. To make change happen, theory needs to be transformed into practice, and actors need to believe in the message. It is sometimes thought that the risk of using antibiotics in animal production is antibiotic residues in meat and other animal-derived food (22). However, this risk is managed by regulations on withdrawal periods before slaughter and harvesting of eggs or milk from treated animals. The real risk is the overuse of antibiotics in animal production, and compelling scientific evidence emphasizes the need to take action (2, 23, 24).

Farmers and veterinarians have been identified as key players in work to contain ABR in the animal sector (25). In poultry production, methods to decrease the need of antibiotics include biosecurity, hygiene, management, vaccine, and probiotics (26, 27). Previous studies from Europe and the US have concluded that veterinarians in pig, cattle and dairy farming in general supported the reduction of antibiotic use (25), but some veterinarians felt pressured by farmers, feed suppliers and others to use antibiotics (25). A UK study found several factors that significantly influenced veterinarians in their decision-making process, including type of case, client relationship, colleagues in the same practice, time pressure, habit, willingness to pay and confidence in the farmer (28). US veterinarians were influenced by expectations and obligations to e.g., other veterinarians, clients, consumers, pharmaceutical companies, and regulatory bodies (29). Hence, it seems important that the various stakeholders agree on how to breed and raise animals without extensive use of antibiotics, in order to support veterinarians in prescribing antibiotics restrictively.

Studies have shown that stakeholders in animal production may believe they use less antibiotics than others (25). This could also be the case for our informants, who expressed views about “others” using more antibiotics. However, statistics on antibiotic use in animals confirm that the overall antibiotic usage in Sweden is low in international comparison (10).

The farmers in our study said that they did not need to reduce the use of antibiotics, it was already zero or close to zero. They were more eager to describe their daily efforts to keep their animals healthy, possibly reflecting policy efforts to focus on preventive animal health work. Farmers in other countries in Europe and the US acknowledge the need for reducing antibiotic use, but some believe in the necessity of antibiotics for a good profit in animal production (25, 30, 31). To change farmers' perceptions and practices, it has been suggested that veterinarians could play a role as sources of information and to facilitate learning processes (25, 30). In the present study informants highlighted the close network of farmers and veterinarians and said this was a facilitating factor. The opposite situation was described among sheep and beef farmers in the UK who reported taking decisions themselves regarding when to treat their animals with antibiotics (31). Industry veterinarians in the present study seemed to play a role as a link between veterinary authorities and the farmers. In addition, the farmers felt involved in the development of production methods. This suggests that farmers were not only passive receivers of guidelines. On the contrary, there seems to have been an exchange of information among stakeholders. Farmers were sufficiently educated to understand the background of new management methods and they were given the opportunity to contribute with their knowledge. Implementation research shows that passive distribution of guidelines is ineffective and that active measures are more successful (32, 33).

With an increasing global population and thereby increased demand for food, poultry farming has provided meat at a low cost in high-density poultry farms (26). However, a problem is that broilers in many regions of the world are raised in overcrowded stables, with poor hygiene and a high risk of bacterial infections, and low doses of antibiotics are routinely given to manage infections (26, 34, 35). The informants in our study hoped that Sweden could act as role model by showing that large-scale poultry farming without extensive antibiotic use is possible. The global nature of the poultry industry, with its reliance on imported breeding material, is one aspect of the perception that ABR comes from outside Sweden. The experience of importing ESBL with day-old chicks and successful efforts to reduce this risk (11) may have contributed to this view.

Products must be sold, and consumers' preferences affect how food is produced and on how antibiotics are used in animal production (25, 29). Price is a major factor influencing choice. The informants in our study expressed concerns about consumers preferring cheaper meat produced outside Sweden. High prices of recommended foods were identified as a key barrier to buying sustainable food (36) and price was the main limiting factor for buying organic poultry meat (37). Another factor of influence is the country of production. Preferences for indigenous chicken meat and egg were high among consumers in Kenya (38) and consumers in Finland preferred broilers produced in Finland (21). Consumers from five different countries, Germany, France, Denmark, China, and Thailand, preferred food from economically developed rather than less developed countries (39). Other factors that may influence choice is animal welfare and organic production methods. The informants in our study presented the Swedish large-scale production of poultry meat with focus on animal health as a sustainable alternative, which they hope will continue to be the choice of customers.

To decrease the need of antibiotics globally, new animal health management methods must be introduced in many countries (26, 27). This may increase production costs, which has been identified as a hindering factor for reducing antibiotic use and for reducing capacity for reinvestment in farm buildings (25). However, efforts in the animal sector alone are not enough to contain antibiotic resistance.

ABR can be looked upon from different perspectives and accordingly be described as different problems in need of different strategies (40). One Health can be used to conceptualize and address strategies concerning ABR (40). The One Health approach means that measures must be taken in human, animal and environmental sectors and that actions should be coordinated (2, 5). According to our study informants the One Health approach is implemented at policy level but not in practice. They felt that containment of ABR in Sweden has primarily engaged the three sectors separately. For countries in the process of developing their ABR action plans, a One Health approach is most likely helpful.

### Methodological Considerations

Trustworthiness is of major importance in all research. In this study we used the criteria developed by Lincoln and Guba to ensure high quality of data and research process (41, 42). To meet the criterion of credibility we recruited stakeholders at different levels in the poultry sector, with different experiences, to gain a broad view of perceptions. The analysis was well-structured and carefully performed. Quotations from the text were used to demonstrate confirmability. Transferability must be judged by the readers, the description of data collection and analysis as well as background information about the participants will facilitate this. Most of the stakeholders were representative for policymakers and field-level actors in the poultry sector, but several informants also had a wider livestock production perspective. The poultry farmers were recruited via the veterinarians, and it is possible that they had more knowledge and were more motivated to work according to guidelines than farmers in general. The interview with the layer farmer was intended to get a wider picture This farmer had never used antibiotics in farm animals, so this was not an issue for the farmer at all. Instead, the issue was good management, and keeping animals healthy. The number of informants, particularly farmers, was limited due to practical and financial reasons. However, based on the homogeneity of the poultry industry and the close cooperation between industry stakeholders within the Swedish Poultry Meat Association, we believe that the picture presented by the interviews illustrates the main views of the industry. We chose personal interviews, which often give richer material, instead of by telephone, which might have produced more interviews. Our findings are in line with the perceived general opinion in the Swedish livestock sector, and the consistency in responses indicate that our findings reflect the perceptions, knowledge, attitudes, and practices of stakeholders in the broiler production sector.

## Conclusion

The interviewed stakeholders in food-animal production in Sweden were committed to the ABR issue. They stated that, in their respective fields, a facilitating factor was that ABR was included in animal health and welfare issues. For the farmers, the most important issue was keeping animals healthy, with daily farm management methods. Further facilitating factors were a close cooperation among stakeholders, common beliefs, and a feeling of belonging to a long tradition of focus on animal health. The stakeholders were proud of the Swedish animal production, but at the same time worried about not being able to sell their products on the international market, as the animal health management system focusing on disease prevention was deemed more expensive than methods using more antibiotics. In showing that intensive broiler production without extensive use of antibiotics is possible, the stakeholders hoped that the Swedish example in this area could serve as a role model for others.

## Data Availability Statement

The datasets presented in this article are not readily available because they consist of in-depth key informant interviews containing sensitive participant information. Due to the small number of persons in this field in Sweden, it may be possible to deduce the identity of the interviewee, which would violate the anonymity agreement with the participants. Requests to access the datasets should be directed to Jaran Eriksen, jaran.eriksen@ki.se.

## Ethics Statement

The studies involving human participants were reviewed and approved by The Stockholm Regional Ethics Board (Ref number: 2017/1999-31). The patients/participants provided their written informed consent to participate in this study.

## Author Contributions

The study was conceptualized by CS and JE. MR and IB developed the methodology and IB did the formal analysis. The analysis was validated by all authors. IB drafted the main manuscript text. SS contributed veterinary expertise and input on the interpretation of the results. All authors reviewed the manuscript.

## Conflict of Interest

The authors declare that the research was conducted in the absence of any commercial or financial relationships that could be construed as a potential conflict of interest.
